# Ocular manifestations of reactive infectious mucocutaneous eruption (RIME) secondary to adenovirus: A case report

**DOI:** 10.1016/j.ajoc.2025.102363

**Published:** 2025-06-07

**Authors:** L.H. Young, S.B. Kim, J.S. Takhar, L.D. Sun, K.M. White, A.F. Omar

**Affiliations:** a Case Western Reserve University School of Medicine. Cleveland, OH, USA; b University Hospitals Cleveland Medical Center, Department of Ophthalmology. Cleveland, OH, USA; c University Hospitals Cleveland Medical Center, Department of Dermatology. Cleveland, OH, USA; d Department of Ophthalmology, Assiut University Hospitals, Assiut, Egypt

**Keywords:** Reactive infectious mucocutaneous eruption (RIME), Conjunctivitis, Adenovirus

## Abstract

**Purpose:**

To present an atypical case of reactive infectious mucocutaneous eruption (RIME) in an adult patient attributed to primary adenoviral infection.

**Observations:**

This is a case describing the clinical course of a previously healthy 34-year old man who presented with acute onset urethritis and bilateral conjunctivitis. His left eye conjunctivitis progressed to pseudomembrane and symblepharon formation within days. His clinical course was marked by progressive worsening of urethritis and formation of mucus membrane ulceration and bullae even after antibiotic treatment and aggressive topical lubrication. He required admission and a multidisciplinary team made the diagnosis of adenovirus-induced RIME. He was treated with intravenous steroids and intravenous cyclosporine. He ultimately recovered with a good visual outcome.

**Conclusions and importance:**

This case demonstrates a rare case of RIME in an adult triggered by an adenovirus infection. Multidisciplinary care is key to diagnosis and treatment.

## 1 Introduction

Reactive infectious mucocutaneous eruption (RIME) is a rare dermatologic condition with ocular and other mucus membrane manifestations. RIME was previously known as *Mycoplasma pneumoniae*-associated mucositis (MIRM). Though MIRM was initially thought to exist on a diagnostic continuum with Stevens-Johnson syndrome (SJS), toxic epidermal necrolysis (TEN), and erythema multiforme (EM),^1^ Canavan et al. first proposed that MIRM was a distinct clinical entity in 2015.^2^ MIRM can be differentiated from SJS/TEN/EM by its infectious etiology and prodrome, preferential involvement of at least two mucous membranes with minimal cutaneous involvement, and mild disease course.^2^, ^3^, ^4^

After the discovery that MIRM could be triggered by other infectious agents, such as *Chlamydia pneumoniae*,^5^^,^^6^ influenza,^7^^,^^8^ rhinovirus,^9^ adenovirus,^10^, ^11^, ^12^ coxsackievirus,^13^ human metapneumovirus,^14^ Epstein Barr virus,^11^ and SARS-COV-2,^8^^,^^15^, ^16^, ^17^ it was renamed RIME.^3^ To the best of our knowledge, the literature reports only three pediatric cases of adenovirus-triggered RIME^10^^,^^11^ and prior published reports only sparsely detail ocular presentation, treatment, and sequelae for RIME.^1^ This case report examines an adult diagnosed with RIME secondary to adenovirus and details the ocular involvement, treatment, and recovery.

## 2 Case report

A 34 year-old healthy African American man with myopia and daily contact lens wear presented to an urgent clinic for two days of left eye redness and dysuria. He reported a recent new sexual contact and subsequently tested negative for gonorrhea/chlamydia. He received one dose of intramuscular ceftriaxone and a course of oral doxycycline and metronidazole. His symptoms persisted and he re-presented to the emergency room with left eye pain, blurry vision, and non-purulent eye discharge, as well as persistent dysuria. He reported his partner also had bilateral eye redness and tearing. Visual acuity was 20/20 (VA) in both eyes (OU) and slit lamp exam was remarkable for 1–2+ conjunctival hyperemia with follicles, mucoid accumulation, 1–2+ bulbar conjunctival injection and scattered subconjunctival hemorrhages. The patient was started on erythromycin ophthalmic ointment 3 times daily to the left eye, instructed to stop PO doxycycline and received a dose of azithromycin to manage concern for sexually transmitted infection etiology. The patient was also started trimethoprim/sulfamethoxazole (TMP/SMX) empirically for suspected urinary tract infection (Table 1).

**Table 1:** Detailed timeline and clinical course.

Time	Symptoms	Treatments	Testing
Day 0	- 2 days of dysuria	1st urgent care visit - IM ceftriaxone. Start doxycycline and metronidazole	(−) G/C
Day 2	- Continued dysuria - OS pain, blurry vision, nonpurulent drainage	1st ED visit - Stop doxycycline - Start azithromycin and TMP/SMX - Start erythromycin ophthalmic ointment	(−) Trichomonas vaginalis, G/C, HIV, syphilis, urine cultures
Day 4	- OS vision 20/60 ph 20/40 - OD involvement begins. - URI symptoms	1st outpatient ophthalmology f/u - Start moxifloxacin drops, cool compresses, PRN artificial tears OS	n/a
Day 6	- Both eyes worse - Raw sensation in mouth	2nd outpatient ophthalmology f/u - Stop moxifloxacin drops - Start on tobramycin-dexamethasone eye drops QID OU. Continue artificial tears, erythromycin ointment	∗Fig. 1 pictures taken
Day 7	- Dysphagia, bleeding oral ulcers - Penile ulcer	2nd urgent care visit - Stop TMP/SMX - Start cephalexin, prednisone 60mg taper	n/a
Day 9	- Oral ulcers, dysphagia, inability to swallow - Penile ulcers, frank hematuria - Improved eye pain and discharge	2nd ED visit. Admitted to hospital - Start IV methylprednisolone 100mg q24h - Stop cephalexin - Continue eye regimen - Supportive care for oral and penile ulcers	**(+) adenovirus nasopharyngeal PCR** (−) HIV, syphilis, G/C, respiratory virus panel, atypical pneumonia panel, urine cultures, mucosal HSV PCR and fungal smear/culture ∗Fig. 3, Fig. 4 pictures taken
Day 10	- Stable oral and URI symptoms - Worse dysuria	- Continue IV methylprednisolone - Continue supportive care for eyes, oral, and GU tract	**(+) adenovirus nasopharyngeal PCR** (−) COVID
Day 12	- Worse dysuria, oral pain, GU lesions	- Stop IV methylprednisolone - Start IV cyclosporine 2mg/kg BID	(−) HSV swab of oral mucosa ∗Fig. 2 picture taken
Day 17	- Improving oral, GU symptoms	- Transition to PO cyclosporine	**(+) adenovirus nasopharyngeal PCR**
Day 19	- Improving eye and oral symptoms	Discharge from hospital - Cyclosporine taper	n/a
Day 24	- OS 20/50 ph 20/40	3rd outpatient ophthalmology f/u - Stop tobramycin-dexamethasone drops. Continue lubrication	n/a
Day 59	- Both eyes back to 20/20 Improved ocular surface	4th outpatient ophthalmology f/u	n/a
Day 108	- OS residual mild subconjunctival scarring	Last ophthalmology f/u	n/a

**Fig. 1:**
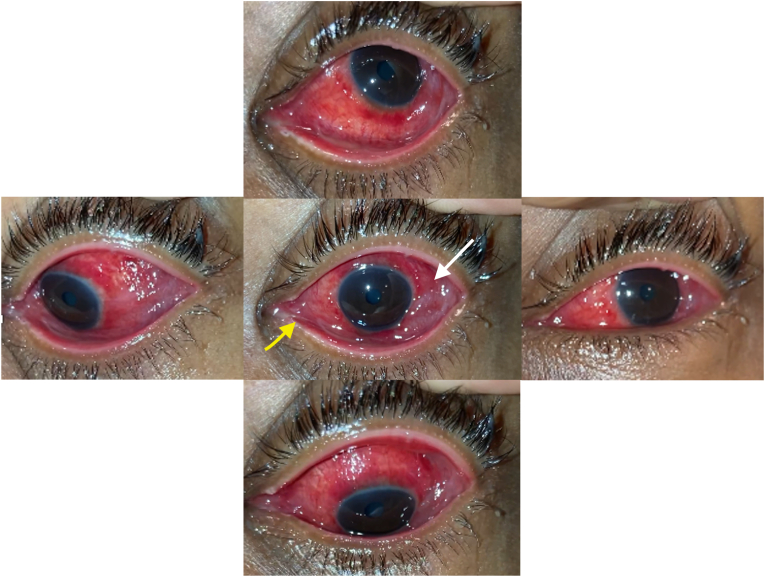
Composite external photo of the left eye in different positions of gaze on day 6. Note the extensive conjunctival injection and chemosis (white arrow) and mild mucoid discharge (yellow arrow). (For interpretation of the references to colour in this figure legend, the reader is referred to the Web version of this article.)

Despite change in management, the patient experienced progression in symptoms with left eye VA decline to 20/60 (pinhole to 20/40) on day 4, new diffuse conjunctival injection, chemosis, upper eyelid pseudomembrane, early lower lid symblepharon formation, and subepithelial corneal infiltrates (Fig. 1). The right eye also developed inferior forniceal conjunctival injection. Bilateral preauricular lymphadenopathy was noted. Due to the clinical picture, an aggressive form of adenoviral conjunctivitis was suspected, and he was started on tobramycin-dexamethasone eyedrops four times a day to both eyes for the interval development of subepithelial infiltrates. He continued his course of oral TMP/SMX (Table 1).

**Fig. 2:**
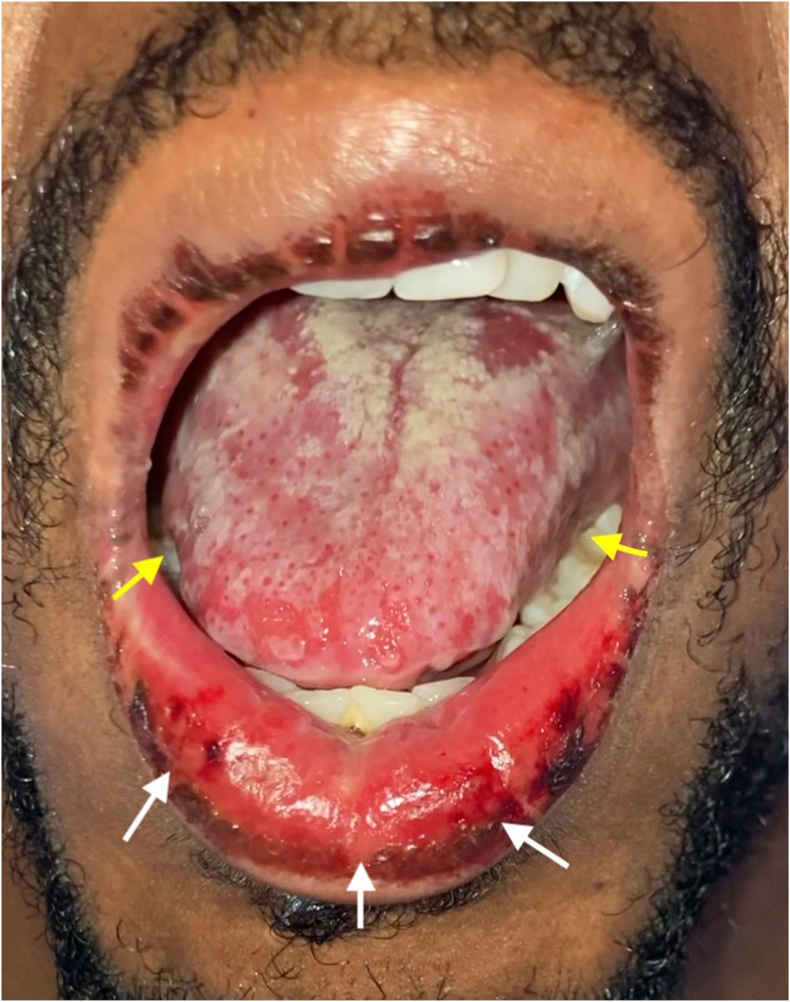
Representative photograph of beefy erosions on lower lip (white arrows) and lateral tongue oral ulcers (yellow arrows). Picture taken on day 12. (For interpretation of the references to colour in this figure legend, the reader is referred to the Web version of this article.)

**Fig. 3:**
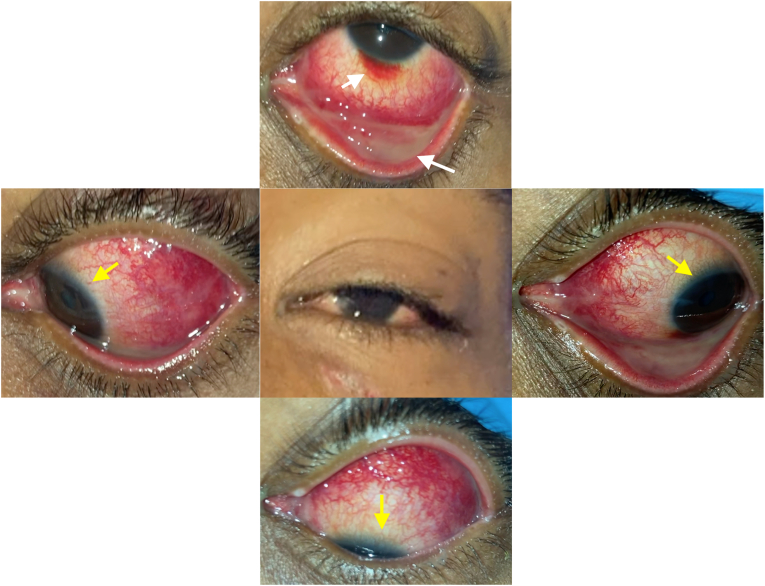
Composite external photo of the left eye in different positions of gaze on day 9. Note the more exaggerated inflammatory response - subconjunctival hemorrhage, pseudomembranes - in the conjunctival fornices (white arrows) compared to the limbus (yellow arrows). (For interpretation of the references to colour in this figure legend, the reader is referred to the Web version of this article.)

**Fig. 4:**
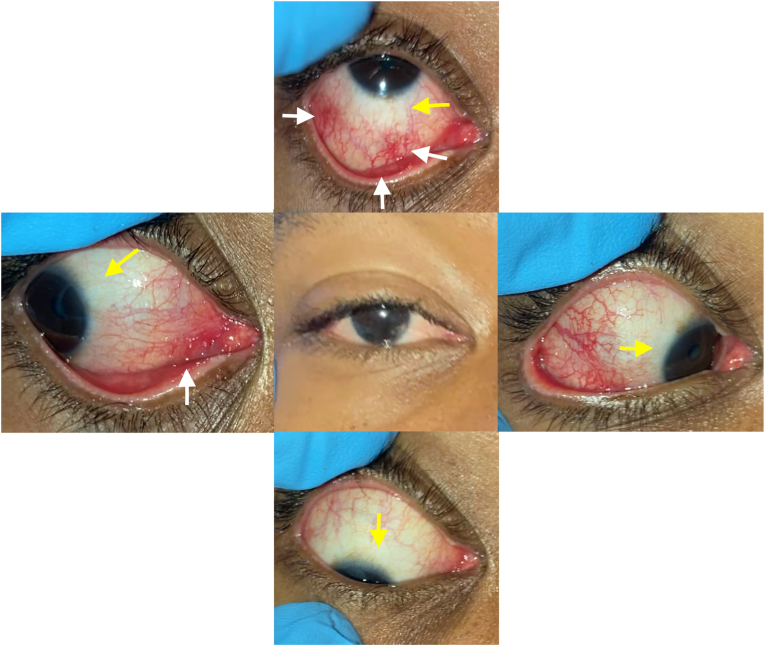
Composite external photo of the right eye in different positions of gaze on day 9 Note the more exaggerated inflammatory response (dilated conjunctival vessels, pseudomembranes, white arrows) in the conjunctival fornices compared to the limbus (yellow arrows). (For interpretation of the references to colour in this figure legend, the reader is referred to the Web version of this article.)

On day 7 after initial symptom onset, the patient represented to an urgent care center with worsening dysphagia, and bleeding oral ulcers (Fig. 2). A drug reaction to TMP/SMX was suspected, so he stopped this medication and started on prednisone 60mg taper. Due to progressive worsening of dysphagia and increased difficulty swallowing, on day 9, the patient re-presented to the emergency room and was subsequently admitted due to poor oral intake. Though the dysphagia was worse, the patient stated he had notable improvement in ocular pain and eye discharge (Fig. 3, Fig. 4). An extensive infectious and inflammatory workup was conducted, which was positive for adenovirus by nasopharyngeal swab polymerase chain reaction (PCR). The rest of the extensive testing, including HIV, syphilis, gonorrhea, chlamydia, respiratory virus panel, atypical pneumonia panel, HSV PCR of mucosal swabs, urine cultures, and fungal culture/smear of mucosal lesions was negative. At this point, the diagnosis of RIME was suggested by the dermatology service (Table 1).

All systemic antibiotics were stopped and intravenous methylprednisolone was started. On day 12, after 3 days of IV methylprednisolone, the patient requested the IV methylprednisolone be switched to cyclosporine due to lack of improvement. By day 17, his pain and PO intake improved. While admitted, the patient required regular debridement of reaccumulated pseudomembrane material and to relieve ocular discomfort. He was continued on a topical regimen of tobramycin-dexamethasone drops four times a day, erythromycin ointment at night, and preservative free artificial tears four times a day in both eyes. The patient's symptoms continued to improve with immunosuppressive therapy. He was discharged on a cyclosporine taper. Two months after discharge his vision had returned to baseline, and slit lamp exam was only notable for a small conjunctival bullae. He was slowly tapered off tobramycin/dexamethasone and maintained on regular lubrication. Three months later, his vision continued to be at baseline and he only had residual mild subconjunctival scarring (Table 1).

## 3 Discussion

This is a rare case of adenovirus-associated RIME in a previously healthy adult man. After conducting a literature review on March 18, 2024 utilizing PubMed and Google Scholar, using the key words “RIME” and “adenovirus” we found reports of three patients with RIME triggered by adenovirus in children^10^, ^11^, ^12^ but no reports of RIME triggered by adenovirus in adults. In the most recent retrospective cohort study of RIME to date of 50 pediatric patients, Pan and Hussein find the average age at presentation is 11.6 years old and predominantly Caucasians (76 %) and males (66 %) are affected.^11^ The most common trigger in their cohort was Mycoplasma pneumoniae, though other causes in their cohort included adenovirus, influenza, Epstein-Barr virus, and SARS-Cov-2. RIME is usually preceded by a prodrome of respiratory infection symptoms, most commonly a dry or productive cough, fever, and malaise. Mucocutaneous eruptions typically occur 8 days later.^2^^,^^18^ The oral mucous membranes are most commonly affected with vesiculobullous lesions being the dominant morphology. Patients can develop oral erosions and hemorrhagic crusting of the lips.^2^^,^^19^^,^^20^ Ocular mucous membranes are the second most common mucositis site in RIME, closely followed by urogenital involvement.^2^^,^^21^

The diagnosis of RIME in this case was not entirely clear at first and took nine days, partially because of the unusual presentation in an adult patient. It was also confounded by the possibility that the patient had Stevens Johnson Syndrome (SJS), especially given the use of antibiotics (particularly TMP-SMX) that are associated with SJS. However, the dermatology service thought SJS was less likely as an initial diagnosis given that he had developed symptoms in two mucus membrane sites (urethra and eye) prior to the initiation of TMP-SMX. The possibility that the subsequent antibiotics contributed to the development of SJS was also considered. But ultimately, given his severe mucositis, relatively sparse cutaneous findings (ie, skin lesions involving less than 1 % of body surface area), lack of a Nikolsky sign, and the positive adenovirus PCR, SJS was thought to be unlikely and RIME was thought to be a more parsimonious diagnosis.

The pathophysiology of RIME is not completely understood, especially in adenovirus-associated RIME. However, in the more commonly reported mycoplasma-induced RIME, proposed theories include immune complex deposition in mucosa and skin which activate complement or phagocytic cells,^1^ polyclonal activation of B cells and plasma cells, genetic suspectibility, or combination of infection downregulating cytochrome p450 enzyme expression which decreases the threshold for a medication to cause a drug reaction.^3^ These cases are more frequently described in children, though a case series of 11 mycoplasma-induced RIME in 2022 did include 5 adults,^22^ suggesting that adult cases of RIME are perhaps underdiagnosed and underreported.

Possible strategies to decrease the time to diagnosis to this case could have been conjunctival scrapings for adenovirus,^23^^,^^24^ or a PCR of a conjunctival swab for adenovirus.^25^^,^^26^ These are more typically used for the diagnosing adenovirus keratoconjunctivitis but could be considered as an adjunctive test for adenovirus-induced RIME. Ultimately, it was likely the multidisciplinary nature of this patient's care and treatment that led to his eventual diagnosis and good outcomes.

For the most part, RIME case reports have been published in the dermatology literature^10^^,^^12^ and describe bilateral conjunctivitis, conjunctival injection, and eyelid swelling^1^ but generally do not provide much detail about ocular findings or long-term ocular sequelae, though ocular involvement is common^2^ and ocular complications can be the most serious sequelae of RIME.^3^ The ophthalmology-specific literature on RIME is sparse.^1^^,^^12^^,^^22^^,^^27^ Haseeb et al. summarized 36 articles and found that RIME patients often have bilateral ocular involvement, mostly involving the conjunctiva, followed by ocular discharge, conjunctival epithelial defects, pseudomembranes, and corneal epithelial defects.^1^ No forniceal involvement has been previously reported, which was a unique feature of the conjunctivitis in our case.

As RIME is so rare, there are currently no formal treatment guidelines. Ramien et al. describe a two-pronged treatment approach that uses antimicrobial therapy (particularly macrolides) to address the underlying infection and immunomodulatory therapy to treat the affected mucus membranes.^3^ In regards to specific immunomodulatory agents, cyclosporine,^28^^,^^29^ oral prednisone,^28^ intravenous immunoglobulins,^1^ etanercept,^30^ and intravenous prednisolone^31^ have been used in both pediatric and adult patients. The mean time between diagnosis to symptom resolution is approximately 2 weeks with the above-mentioned therapies.^1^ About 8–38 % of patients develop recurrent episodes, but the risk of recurrence is significantly less with an infectious prodrome and non-oral mucositis.^11^

Treatments for ocular manifestations of RIME are also not well characterized, but typically involve lubrication, topical steroid (predominantly prednisolone acetate 1 %) and antibiotic drops (fluoroquinolones, macrolides, and choramphenicol), and amniotic membrane transplants^1^ in severe cases. Gise et al. propose a grading system for ocular involvement, then recommend a stepwise ocular treatment algorithm based on the grading.^12^ Mild disease is described as minimal ocular involvement, moderate disease is defined as less than half of the lid margin staining or conjunctival staining with no corneal involvement, and severe disease is defined as more than half of lid margin staining bilaterally, corneal epithelial defects, or areas of >1cm of conjunctival staining.^12^ Mild cases are treated with prophylactic lubrication, moderate cases add antibiotic/steroid eye drops four times daily and antibiotic/steroid ointment nightly. Finally, treatment of severe disease encompasses all the prior treatments and adds amniotic membrane transplant or temporary sutureless amniotic membrane transplant (Prokera, BioTissue, Miami, FL).^12^

## 4 Conclusions

We seek to augment the sparse literature on ocular manifestations of adenovirus-triggered RIME by detailing a rare case in an adult patient. This case was defined by rapid, multisystemic disease progression and emphasizes the need for close follow up with these patients. It also highlights the importance of multidisciplinary approach to treatment, as collaboration between ophthalmology, dermatology, urology, ENT, and infectious disease services was needed to achieve prompt diagnosis and good outcome. We hope this may aid other ophthalmologists and physicians in identifying the ocular and systemic signs and symptoms of RIME, to promote prompt intervention and improved clinical outcomes.

## Intellectual property

We confirm that we have given due consideration to the protection of intellectual property associated with this work and that there are no impediments to publication, including the timing of publication, with respect to intellectual property. In so doing we confirm that we have followed the regulations of our institutions concerning intellectual property.

## CRediT authorship contribution statement

**L.H. Young:** Writing – review & editing, Writing – original draft, Investigation, Data curation. **S.B. Kim:** Writing – review & editing, Writing – original draft, Investigation, Data curation. **J.S. Takhar:** Writing – review & editing, Formal analysis, Conceptualization. **L.D. Sun:** Conceptualization, Data curation, Writing – review & editing. **K.M. White:** Writing – review & editing, Writing – original draft, Data curation. **A.F. Omar:** Writing – review & editing, Conceptualization.

## Patient consent

The patient verbally consented to publication of the case.

## Research ethics

We further confirm that any aspect of the work covered in this manuscript that has involved human patients has been conducted with the ethical approval of all relevant bodies and that such approvals are acknowledged within the manuscript.

Verbal consent to publish potentially identifying information, such as details or the case and photographs, was obtained from the patient(s) or their legal guardian(s).

## Authorship

The International Committee of Medical Journal Editors (ICMJE) recommends that authorship be based on the following four criteria:

1. Substantial contributions to the conception or design of the work; or the acquisition, analysis, or interpretation of data for the work; AND
2. Drafting the work or revising it critically for important intellectual content; AND
3. Final approval of the version to be published; AND
4. Agreement to be accountable for all aspects of the work in ensuring that questions related to the accuracy or integrity of any part of the work are appropriately investigated and resolved.

All those designated as authors should meet all four criteria for authorship, and all who meet the four criteria should be identified as authors. For more information on authorship, please see http://www.icmje.org/recommendations/browse/roles-andresponsibilities/defining-the-role-of-authors-and-contributors.html#two.

All listed authors meet the ICMJE criteria.  We attest that all authors contributed significantly to the creation of this manuscript, each having fulfilled criteria as established by the ICMJE.

One or more listed authors do (es) not meet the ICMJE criteria.

We believe these individuals should be listed as authors because:

We confirm that the manuscript has been read and approved by all named authors.

We confirm that the order of authors listed in the manuscript has been approved by all named authors.

## Contact with the editorial office

This author submitted this manuscript using his/her account in EVISE.

We understand that this Corresponding Author is the sole contact for the Editorial process (including EVISE and direct communications with the office). He/she is responsible for communicating with the other authors about progress, submissions of revisions and final approval of proofs.

We confirm that the email address shown below is accessible by the

Corresponding Author, is the address to which Corresponding Author's EVISE account is linked, and has been configured to accept email from the editorial office of American Journal of Ophthalmology Case Reports: Ahmed.Omar@UHhospitals.org.

Someone other than the Corresponding Author declared above submitted this manuscript from his/her account in EVISE:

We understand that this author is the sole contact for the Editorial process (including EVISE and direct communications with the office). He/she is responsible for communicating with the other authors, including the Corresponding Author, about progress, submissions of revisions and final approval of proofs.

We the undersigned agree with all of the above.

## Funding

No funding was received for this work.

## Declaration of competing interest

The authors declare that they have no known competing financial interests or personal relationships that could have appeared to influence the work reported in this paper.
